# Mucin granule-associated proteins in human bronchial epithelial cells: the airway goblet cell "granulome"

**DOI:** 10.1186/1465-9921-12-118

**Published:** 2011-09-06

**Authors:** Kimberly L Raiford, Joungjoa Park, Ko-Wei Lin, Shijing Fang, Anne L Crews, Kenneth B Adler

**Affiliations:** 1 Department of Molecular Biomedical Sciences, College of Veterinary Medicine, North Carolina State University, Raleigh, North Carolina, USA; 2 Department of Biochemistry and Biophysics, University of North Carolina at Chapel Hill, Chapel Hill, North Carolina, USA; 3 Department of Medicine, University of California, San Diego, California, USA

## Abstract

**Background:**

Excess mucus in the airways leads to obstruction in diseases such as chronic bronchitis, asthma, and cystic fibrosis. Mucins, the highly glycosolated protein components of mucus, are stored in membrane-bound granules housed in the cytoplasm of airway epithelial "goblet" cells until they are secreted into the airway lumen via an exocytotic process. Precise mechanism(s) of mucin secretion, including the specific proteins involved in the process, have yet to be elucidated. Previously, we have shown that the Myristoylated Alanine-Rich C Kinase Substrate (MARCKS) protein regulates mucin secretion by orchestrating translocation of mucin granules from the cytosol to the plasma membrane, where the granules dock, fuse and release their contents into the airway lumen. Associated with MARCKS in this process are chaperone (Heat Shock Protein 70 [HSP70], Cysteine string protein [CSP]) and cytoskeletal (actin, myosin) proteins. However, additional granule-associated proteins that may be involved in secretion have not yet been elucidated.

**Methods:**

Here, we isolated mucin granules and granule membranes from primary cultures of well differentiated human bronchial epithelial cells utilizing a novel technique of immuno-isolation, based on the presence of the calcium activated chloride channel hCLCA1 (the human ortholog of murine Gob-5) on the granule membranes, and verified via Western blotting and co-immunoprecipitation that MARCKS, HSP70, CSP and hCLCA1 were present on the granule membranes and associated with each other. We then subjected the isolated granules/membranes to liquid chromatography mass spectrometry (LC-MS/MS) to identify other granule associated proteins.

**Results:**

A number of additional cytoskeletal (e.g. Myosin Vc) and regulatory proteins (e.g. Protein phosphatase 4) associated with the granules and could play a role in secretion were discovered. This is the first description of the airway goblet cell "granulome."

## Background

The role of the airway epithelium extends well beyond its function as a physical barrier between external and internal milieu. For example, airway epithelium provides for overall pulmonary homeostasis mediating inflammatory responses to injury, regulates lung fluid balance and anti-oxidant release, and is responsible for clearance of inhaled agents via the mucociliary system [1]. Mucins, the highly glycosolated protein components of mucus, are stored in membrane-bound granules in the cytoplasm of airway epithelial secretory (goblet) cells. When mucins are secreted, a thin layer of mucus forms that protects airways from inhaled pathogens and particulates, which are subsequently cleared out of the airways via mucociliary transport [2,3].

Actual secretion of mucin into the airway lumen occurs by a process of regulated exocytosis involving translocation of granules from the cytoplasm of the goblet cells to the plasma membrane, where they dock and, following fusion of the granule and plasma membranes, release their mucin contents into the airway lumen [4]. While constitutively low levels of secreted mucin are involved in the normal mucociliary clearance mechanism, mucin hypersecretion results in excess mucus in the airways and is a phenotype associated with chronic inflammatory diseases such as chronic bronchitis, asthma, and cystic fibrosis [3,5,6]. Several proteins have been associated with the mucin hypersecretory phenotype, including myristoylated alanine-rich C kinase substrate (MARCKS), calcium activated chloride channel (hCLCA1), and chaperones cysteine string protein (CSP) and heat shock protein 70 (HSP70) [7-9]. However, interactions of these proteins, as well as additional proteins involved in the secretory process, are poorly understood, thus few potential therapeutic targets to control excessive airway mucus secretion have been elucidated.

In this report, we isolated mucin granules and granule membranes from well-differentiated normal human bronchial epithelial (NHBE) cells using a novel technique of immuno-isolation and evaluated whether the above-mentioned proteins (MARCKS, CSP, HSP70 and hCLCA) were associated with the granules via Western blotting, and further expanded our scope to identify the granule-associated proteome in NHBE cells, or the "granulome", using liquid chromatography tandem mass spectrometry (LC-MS/MS) of the isolated granules and granule membranes. The results confirm that the above proteins indeed do associate with mucin granules, along with other cytoskeletal, signaling, and accessory proteins. Interestingly, we also found that MARCKS, CSP, and HSP70 appear to complex with hCLCA1. These results reveal, for the first time to our knowledge, proteins associated with intracellular mucin granules that could be involved integrally in the secretory process. A complete description of this "granulome" certainly can increase our understanding of mechanisms and protein interactions involved in mucin secretion, and suggest potential new functions for these proteins as well as new therapeutic targets for control of mucin hypersecretion in airway diseases.

## Materials and methods

### Cell Culture

Primary culture of NHBE cells in air/liquid interface, a technique that allows these cells to maintain a well-differentiated phenotype, has been described previously [10]. Briefly, commercially available NHBE cells from a single donor (Lonza, Cambridge, MA) were seeded into vented T75 tissue culture flasks at a density of 500 cells/cm^2^. The cells were expanded in growth medium at 5% CO_2 _at 37°C to a confluence of 85-90%, dissociated from the flasks using 0.25% trypsin/EDTA, and frozen in liquid nitrogen as passage-2 cells (2 × 10^6 ^cells/ml).

Air/liquid interface cultures of NHBE cells were established on Transwell^® ^-Clear culture 0.4 μm pore polyester inserts (Costar, Cambridge, MA) thinly coated with rat-tail collagen type I (Collaborative Biomedical, Bedford, MA). Frozen NHBE cells were recovered and seeded at a density of 2 × 10^4 ^cells/cm^2 ^onto the apical surface of the inserts with medium added to the basolateral compartment. The complete medium was composed of a 50:50 mixture of bronchial epithelial growth medium and Dulbecco's modified Eagle's medium containing high glucose (4.5 g/L) and a final concentration of 50 μg/ml gentamicin, 5 μg/ml insulin, 10 μg/ml transferrin, 0.5 μg/ml epinephrine, 6.5 ng/ml triiodothyronine, 0.5 ng/ml human recombinant epidermal growth factor, 0.5 μg/ml hydrocortisone, 50 ng/ml amphotericin-B (Lonza), 0.13 mg/ml bovine pituitary extract, 5 × 10-8 mol/L all-trans retinoic acid, 1.5 μg/ml bovine serum albumin (Sigma, St. Louis, MO), and 20 U/ml nystatin (Ameresco, Solon, OH). Cells were grown submerged in a 5% CO_2 _atmosphere at 37°C, and medium was changed the next day, then every other day until cells reached 90% confluence. At this point, air/liquid interface (ALI) was established by removing the apical medium, thus maintaining cells with medium beneath and no medium on top. The medium below was changed daily for 14 days. Mucin was observed at 14 days in culture and cilia were apparent by 18 days. Experiments were conducted on cells at 21 days in culture, ensuring that the cultures were well differentiated. When treating NHBE cells, the apical surface of the cells was washed in phosphate buffered saline (PBS), pH 7 using gentle agitation for 5 min prior to treatment to remove accumulated mucus.

### Immuno-isolation of mucin granules

Granule immuno-isolations were performed using a modified version of a protocol described by Wu et al. [11]. After treatments, cells were washed in PBS and then collected in isolation buffer (PBS, 1 mM phenylmethyl sulfonyl fluoride, protease inhibitor cocktail 1, phosphatase inhibitor cocktail (Sigma, St. Louis, MO)) using a rubber policemen. The collected cells were lysed by brief sonication, and the lysates were spun at 600 × g for 10 min. The supernatants were added to 1.9 volumes of 86% Percoll, 0.3 M sucrose, 5 mM MOPS (4-Morpholinepropanesulfonic acid), 1 mM EDTA, and 0.2 μg/ml DPPD (N, N'-diphenyl-4-phenylenediamine) (Sigma), ph 6.8, and centrifuged for 30 min at 17,000 × g in a Sorvall Discovery 100S ultracentrifuge (Sorvall, Inc. Newtown, CT). The crude granules were transferred from the bottom of the self-formed gradient into a new tube, diluted with 3 volumes of 0.3 M sucrose containing 2 mM MOPS, 1 mM EDTA, and 0.2 μg/ml DPPD, and centrifuged for 15 min at 2000 × g. The pellet was reconstituted in PBS, incubated with an antibody to gob-5/mclca3 (ortholog to human CLCA1) generated in our laboratory overnight at 4°C on a nutator. The rabbit polyclonal gob-5 antibody used was generated to the mclca3 peptide epitope ESWKAKPEYTRPKLE (Covance, Denver, PA). After incubation, the antibody-granule complex was applied to protein G coated Dynal beads. The beads were washed thoroughly and the complex was eluted with Na-citrate pH 2.5 or loading dye.

### Protein subcellular fractionation

After treatments, cells were washed with ice-cold PBS containing a phosphatase inhibitor (Active Motif Inc, Carlsbad, CA) and then scraped into lysis buffer (50 mM Tris, pH 7.5, 1 mM ethylenediamine tetraacetic acid, 100 mM NaCl, 1 mM phenylmethyl sulfonyl fluoride) using a rubber policemen. The collected cellular mixture was lysed by brief sonication. The lysates were spun at 14,000 × g at 4°C in an Eppendorf 5417R centrifuge (Eppendorf Corp., Hamburg, Germany) for 30 min to separate the cytosolic and membrane fractions. The supernatant was kept as the cytosolic sample while the pellet was resuspended in lysis buffer containing 0.01% Triton-100, dissolved by sonication, and incubated on ice for 30 min. Following incubation, the samples were centrifuged again at 14,000 × g at 4°C for 30 min, and the supernatant separated from the pellet mixture was kept as the membrane fraction.

For preparation of whole cell crude lysates, the disrupted cellular mixture was centrifuged at 15,000 rpm in an Eppendorf 5417R centrifuge (Eppendorf Corp., Hamburg, Germany) for 15 min at 4°C. The supernatant was collected as the whole crude cell lysate. The protein concentrations of all cell lysate samples were quantified by a Bradford assay (BioRad Laboratories, Hercules, CA). Bovine serum albumin (BSA; Sigma) was used as the standard and serial dilutions were made from the initial stock concentration of 400 ng/ml. Absorbance values were determined with a microplate reader system, and the linear regression and protein concentrations calculated by SoftMax Pro data analysis software (Molecular Devices, Sunnyvale, CA).

### Co-immunoprecipitation of protein complexes and Western analysis

Whole cell or mucin granule lysates containing 500-1000 μg/ml total protein were incubated overnight at 4°C with 3-10 μl (20-30 μg) with the indicated antibody. Twenty-five μl of Protein G dynal beads (Invitrogen, Carlsbad, CA) was added to bind the antibody-protein complex for 3 hr. Beads were washed three times with cold PBS, and proteins were eluted with 1× sodium dodecyl sulfate-polyacrylamide gel electrophoresis (SDS-PAGE) sample buffer and boiled 10 min before the proteins were resolved on SDS-PAGE gel. Resolved proteins were transferred to a 0.45 μM nitrocellulose membrane (BioRad, Hercules, CA), blocked with 5% skim milk, and either mouse anti-MARCKS (Millipore, Bedford, MA), rabbit anti-CSP, mouse anti-HSP70 (Abcam, Cambridge, UK), goat anti-hCLCA1 (Imgenex, San Diego, CA) or rabbit anti-mclca3 antibody was used as the primary antibody to probe the membranes.

Visualization of the proteins occurred after probing with the secondary horseradish peroxidase-conjugated antibodies using an enhanced chemiluminescence kit (Chemicon, Buckinghamshire, UK) followed by exposure to film. Densitometry was analyzed by Labworks image acquisition and analysis software (UVP Inc, Upland, CA).

### Ultrastructural Immunohistochemistry

Well differentiated cell cultures were fixed on the Transwell insert with 4% formaldehyde: 1% glutaldehyde in phosphate buffer. In mucin granule membrane preparations, the granule membranes were fixed in the magnetic bead slurry. The tissue samples were embedded in Spurr resin, cut into ultrathin sections, and placed on stainless steel grids. Grids were blocked in 10% fetal bovine serum (FBS) in PBS for 15 min at room temperature followed by a 5 min wash in 0.5% BSA in PBS. Primary antibody treatment of the grids was done overnight at 4°C on a nutator, after which the grids were washed repeatedly for one hr in 0.5% BSA in PBS, and probed with gold labeled secondary antibody for 2 hr at room temperature. The appropriate whole molecule IgG was used as the primary antibody negative control. The grids were washed in PBS repeatedly over a 1 hr period, dried quickly, post-stained with uranyl acetate, and examined with a FEI/Philips EM 208S transmission electron microscope. The pan mucin 17Q2 antibody [12] was used as a positive control to identify intact mucin granules.

### Liquid chromatography tandem mass spectrometry (LC-MS/MS)

Protein bands separated on a 1-Dimensional (SDS-PAGE) were excised, dried with solvent, extracted, and treated with hydroxyethyl disulfide as a thiol blocking reagent under alkaline conditions at 60°C. The extracted peptides were reduced nearly to dryness under a stream of air prior to trypsin digestion in 50 mM ammonium bicarbonate pH~7.8. Samples were then incubated overnight at 37°C before analysis by LC/MS.

Peptides were analyzed by reverse phase HPLC with electrospray ionization mass spectrometry. Separations were achieved with a C18 HPLC column (Phenomenex Jupiter Proteo: 150 mm × 0.50 mm I.D., 4 um particle size, 90A pore size) and a mobile phase operated with a programmed gradient with 50 mM acetic acid and acetonitrile. The instrument used for the analysis was a Thermo Surveyor HPLC coupled with a Thermo LTQ ion trap mass spectrometer. The mass spectrometer was operated in positive ion mode with an electrospray ionization (ESI) source. The mass spectrometer was operated in data dependent MS/MS scan mode scanning from m/z 420-2000 and collecting MS/MS spectra on the four most abundant ions in each scan.

### Protein database searching

The acquired MS/MS spectra for each sample were searched using the BioWorks 3.1 SR1 SEQUEST algorithm (Thermo Electron, San Jose, CA) against the human nonredundant database. The nonredundant database was downloaded from the National Center for Biotechnology Information (NCBI) website. The nonredundant database was used for initial protein identification for tandem mass spectral data acquired in the ICR cell as well as the linear trap. Evaluation of total protein coverage was done by creating a protein subset database consisting of *Homo sapiens *proteins only. Database searching parameters assumed proteolysis was performed using trypsin with the possibility of one internal cleavage residue. Searches were performed with trypsin specified as the enzyme with an allowance for up to two missed cleavage sites. Searches from replicates within an experiment were combined to generate a comprehensive list of peptides and proteins identified in a particular experiment. Acceptance levels for positive peptide identification were determined using cross-correlation scores (Xcorr). These scores aid in the determination of true positives, with higher scores increasing confidence in correct identifications. The minimum acceptable Xcorr for identified peptides was 3.0 [13,14].

### Statistical analysis

Replicate experiments were performed for each concentration of reagents assayed. All reagents used in treating the cells were examined for cytotoxicity by measuring the total release of lactate dehydrogenase from the cells and experiments were performed at non-cytotoxic concentrations.

## Results

### Localization of hCLCA1 in NHBE cells via ultrastructural immunohistochemistry (ITEM)

**Figure 1:**
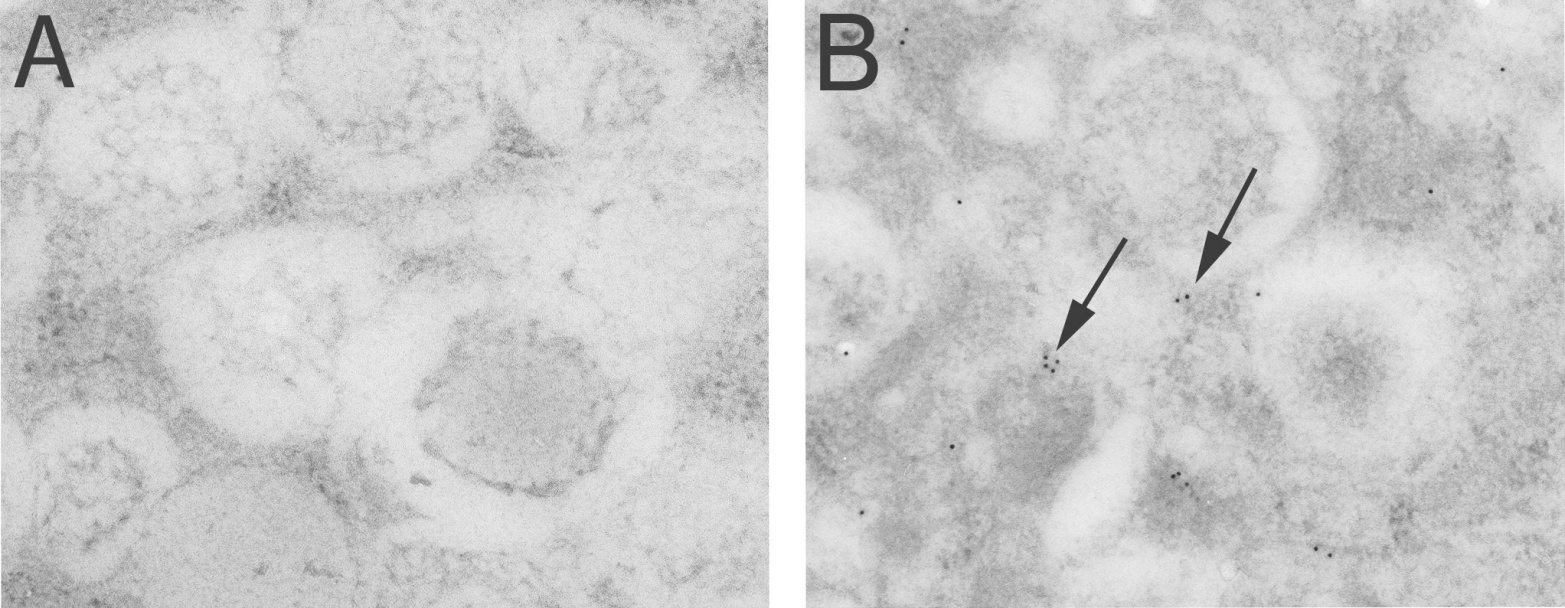
**Association of hCLCA1 with mucin granules within NHBE cells**. Ultra-thin sections of NHBE cells cultured on Transwell^® ^inserts were evaluated by ultrastructural immunohistochemistry to elucidate the subcellular distribution of hCLCA1. Tissue sections were incubated with primary rabbit anti-mclca3 antibody followed by incubation with 12 nm gold-labeled goat anti-rabbit secondary antibody. CLCA1 appears to be localized in proximity to the mucin granules (arrows, B). Negative control using rabbit IgG as the primary antibody; little if any background staining is observed (A). Magnification is at 70Kx.

ITEM of well-differentiated NHBE cells was used to examine the subcellular distribution of hCLCA1. Tissue sections were incubated with primary rabbit anti-mclca3 antibody followed by incubation with 12 nm gold-labeled goat anti-rabbit secondary antibody. hCLCA1 appears to localize at mucin granules membranes (Figure 1B). There was little if any background staining seen in the negative controls in which a non-specific IgG was substituted for the secondary antibody (Figure 1A).

### Validation of immuno-isolation method of mucin granule preparation via TEM and ITEM

**Figure 2:**
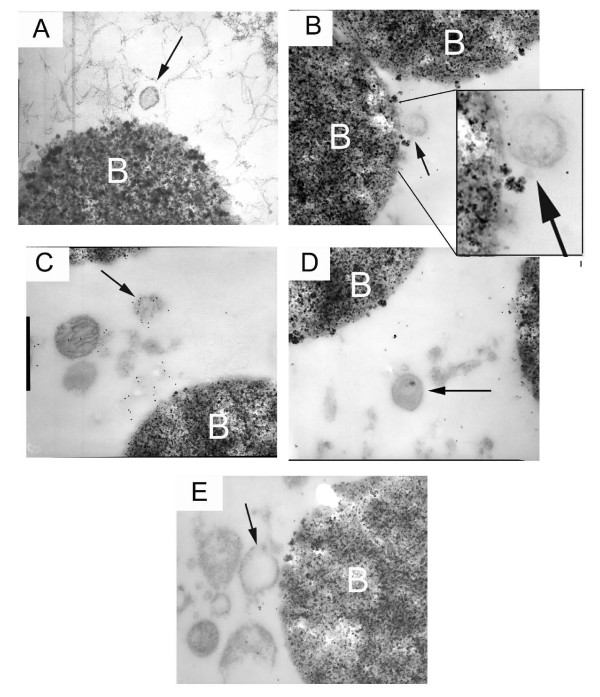
**Ultrastructural analysis of mucin granules isolated from goblet cells of well-differentiated NHBE cells in culture**. Primary antibody incubations were followed by 12 nm gold-labeled secondary antibody. Gold appears as black dots indicating the presence of the primary antibody. A) Mucin granule membranes (arrows) near a magnetic dynal bead (B); B) hCLCA1 localized to mucin granule membranes as demonstrated by gold-labeled immunostaining; C) positive control: gold-labeled pan mucin antibody (17Q2) shows the presence of mucin within the granules; D) Rabbit IgG negative control; E) Mouse IgG negative control. Magnification is at 40Kx.

To verify that the immuno-isolation technique was indeed isolating granules and granule membranes, standard TEM and ITEM were utilized. Figure 2A shows an intact mucin granule membrane isolated by this technique, while Figure 2B demonstrates the presence of hCLCA1 associated with these membranes via gold-labeling. The positive control used 17Q2 (mouse anti-mucin) as the primary antibody, further verifying that these structures are indeed mucin granule membranes (Figure 2C). The negative rabbit and mouse IgG controls are illustrated in Figures 2D and 2E, respectively.

### Association of MARCKS, CSP, and HSP70 with mucin granule membranes

**Figure 3:**
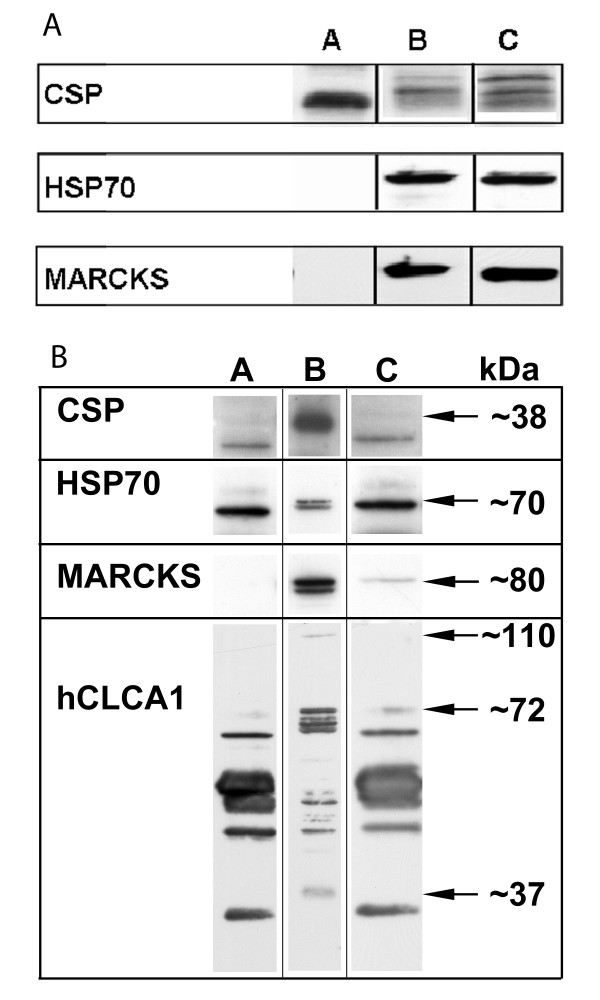
**A) Western blot analysis of mucin granule immuno-isolations reveals that CSP, HSP70, and MARCKS are associated with the granule**. Mucin granules were isolated as described, and isolated granules separated from other whole cell organelles through differential centrifugation in a 86% Percoll gradient and targeted by incubation with the rabbit-anti-mclca3 antibody. Immuno-isolation blots were probed with anti-CSP, anti-HSP70, and anti-MARCKS in unstimulated (Lane C) and PMA-exposed (Lane B) well-differentiated NHBE cell. Cells were exposed to 100 nM PMA for 15 min. Whole molecule rabbit IgG was the negative control used for the immuno-isolations (Lane A). B) CSP, HSP70, MARCKS, and CLCA are associated in NHBE cells. Immunoprecipitation of MARCKS from whole cell lysates followed by immunoblotting for CSP, HSP70, and hCLCA1, in NHBE cells indicates that these proteins appear associated with each other (Lane C). Whole cell lysates (Lane B) also show the presence of these proteins. Immunoblotting with anti-MARCKS antibody was the positive control. Whole molecule rabbit IgG was the negative control used for the immuno-isolations (Lane A).

MARCKS [7], CSP, and HSP70 [9] are reportedly linked to mucin secretion in airway epithelial cells, so we evaluated whether or not these proteins associate with membranes of mucin granules using Western blot analysis. Granules isolated from well differentiated NHBE cells were separated from other whole cell organelles through differential centrifugation in an 86% Percoll gradient, 0.3 M sucrose, then specifically targeted by incubation with a rabbit-anti-mCLCA3 antibody, the mucin granule membrane biomarker. Immuno-isolation blots were probed with anti-CSP, anti-HSP70, and anti-MARCKS antibodies. As illustrated in Figure 3B, MARCKS, CSP, and HSP70 all appear to associate with mucin granule membranes. Whole molecule rabbit IgG was the negative control.

### CSP, HSP70, and hCLCA1 interact with MARCKS in NHBE cells

Since MARCKS, HSP70, CSP, and hCLCA1 all associate with mucin granule membranes, we addressed whether or not they also may associate with each other. As illustrated in Figure 3B, immunoprecipitation of MARCKS from NHBE whole cell lysates followed by detection with anti-CSP, anti-HSP70, and anti-hCLCA1 antibodies in NHBE cells indicates that CSP, HSP70, and hCLCA1 appear to all associate with MARCKS (and thus directly or indirectly with other). Immunoblotting with anti-MARCKS antibody was the positive control for these experiments.

### Additional mucin granule membrane associated proteins identified by LC-MS/MS

Mucin granule membranes isolated as described above were eluted from magnetic beads in SDS sample buffer, boiled for 5 min, and separated by SDS-PAGE. Multiple bands of different molecular sizes were excised from the gel and processed through LC-MS/MS. The band sizes were chosen so as not to include MARCKS, CSP, and hCLCA1 which have already been identified as granule-associated via Western blotting, and their high content could mask additional proteins of similar size. Table 1 shows the proteins identified via LC-MS/MS of the granule/granule membrane preparations, limited to those proteins with an X-corr ≥ 3.0. For proteins known to be related to exocytosis and secretion, we lowered the X-corr to ≥ 2.0; these proteins are indicated by an asterisk in Table 1. The majority of these proteins appear to be cytoskeletal or regulatory. A full listing of proteins with an X-corr ≥ 2.0 is included in additional file 1, Table S1.

**Table 1:** Mucin granule membrane associated proteins identified by LC-MS/MS)

Protein name	Xcorr	NCBI Protein Accession Number
***Cytoskeletal structure-related proteins***		
**Myosin, heavy chain 9**	5.665	29436380
**Gelsolin**	5.283	4504165
**Cytokeratin 9**	4.698	435476
**Keratin 1**	4.684	17318569
**novel protein similar to annexin A2 (ANXA2)**	4.318	12314197
**Anterior gradient 2**	4.116	68012756
**Mutant beta-actin**	4.084	28336
**Annexin A2**	3.904	16306978
**Arp2/3 complex 16 kDa subunit 2**	3.761	33150554
**Tropomodulin 3**	3.632	6934244
**Similar to beta-actin**	3.502	37546764
**Calmodulin 1 (phosphorylase kinase, delta)**	3.458	30583815
**Actin-like protein**	3.294	62421162
**Myosin regulatory light chain MRCL2**	3.259	15809016
**Myosin regulatory light chain MRCL3 variant**	3.259	62896697
**Keratin 6 irs**	3.206	15618995
**Keratin, type II cytoskeletal 3 (Cytokeratin 3)**	3.206	125098
**Keratin 6A**	3.094	30584049
**Keratin 19; keratin, type I cytoskeletal 19; keratin, type I, 40-kd**	3.024	24234699
**Myosin VC**	2.127*	9055284
**Syntaxin 11**	2.157*	3248918
***Regulatory proteins & enzymes***		
**Heat shock 70 kDa protein 5**	4.516	16507237
**Hydroxyacyl-Coenzyme A dehydrogenase/3-ketoacyl-Coenzyme A thiolase/enoyl-Coenzyme A hydratase (trifunctional protein), alpha subunit, isoform CRA_b**	3.388	119621109
**Parkin isoform**	2.403*	20385800
**Protein phosphatase 4, regulatory subunit 2**	2.057*	28372531
**SI:zC214P16.4 (novel protein similar to human protein phosphatase 1)**	2.186*	27884151

* = proteins with Xcorr ≥ 2.0 ≤ 3.0 previously implicated in exocytosis

## Discussion

The aim of the studies described in this report was to identify proteins associated with mucin granules within human airway goblet cells that may play a role in regulated exocytosis. To accomplish this, we utilized a method of subcellular fractionation similar to one used in proteomic analysis of intracellular complexes and organelles, including endothelial membrane rafts [15], neutrophil secretory vesicles [16], and insulin secretory granules [17]. However, a complication of this method arises from the presence of contaminating subcellular fragments that settle in the same density gradient as the target. Thus, we went on to utilize a two tiered approach to subcellular fractionation, which we call "immuno-isolation", in which an antibody specific to the target organelle, in this case an antibody against the known mucin granule membrane-associated protein hCLCA1 (alias Gob-5), is used to further purify the isolates.

Immunoblotting lysates from well-differentiated normal bronchial epithelial cells with a rabbit polyclonal anti-mclca3 antibody identified protein fragments sized at 110, 72, and 40 kDa. Furthermore, immunoprecipitation with the mclca3 antibody followed by analysis with a hCLCA1 specific antibody verified the previous results. These sizes are similar to what has been reported in all other CLCA homologues thus far [18-20]. Therefore, the biochemical results are consistent with the proposed general model of CLCA protein structure and processing (reviewed in [21]).

Although the exact function of CLCA1 in airway goblet cells has not been fully elucidated, certainly the murine clca3 is a granule-associated protein and thus can be used as a biomarker. Human calcium-activated chloride channel and its murine ortholog, mclca3 (alias Gob-5) have been shown to be associated with goblet cell hyperplasia and mucus overproduction [6,8]. Subsequent bioinformatics analysis and immunoprecipitation experiments from the same group [22] identified mclca3/hCLCA1 as a strongly associated mucin granule protein [23,24]. Immune transmission electron microscopy using gold-labeled secondary antibody staining identified mclca3 associated with mucin granule membranes of gastrointestinal, respiratory, uterine goblet cells and other mucin-producing cells [18] thus, it has been used as a biomarker in mucin granule isolations [25]. More recent studies have suggested that hCLCA1 could actually be a secreted protein, rather than a functional channel, most likely a regulator of chloride channels [23,24].

A related finding of interest in this study was that hCLCA1 binds MARCKS in a complex with CSP and HSP70. This is a novel finding that requires additional analysis, but it supports the above idea that hCLCA1/mclca3 is a soluble protein, likely a regulatory subunit, rather than a channel. The appearance of the 40 and 110 kDa fragments of the protein in the cell lysate rather than in the membrane fraction (Figure 3B) also supports the concept of it being a soluble protein. Studies done by Gibson et al. determined that hCLCA1 and mclca3 proteins were secreted in bronchial alveolar lavage fluids from asthmatic patients and ovalbumin challenged mice [23] as fragment variants of these proteins. Furthermore, a CLCA family member, mclca1, was shown to directly interact with a large conductance potassium channel β subunit when co-transfected into HEK293 cells, which upregulated the calcium sensitivity and evoked a larger calcium activated chloride current than when it was transfected alone [23]. It is tempting to speculate that the role of hCLCA1 in mucin granule exocytosis is regulation of the calcium influx that is well established in exocytosis events. While this does not directly address the hCLCA1 interaction with MARCKS, it does provides a possible mechanistic role for hCLCA1 in mucin secretion

Ultrastructural analysis of the isolated mucin granule membranes revealed that both intact and fragmented membrane pieces were isolated by our methods. A more targeted TEM view with both the 17Q2 mucin and mclca3 antibodies labeled with gold particles verified our findings. 17Q2 has been used extensively to measure mucins in ELISA and immunocytochemistry [12,26]. Gold beads were observed congregating around the Dynal beads, showing the affinity of the Protein G dynal beads with the mclca3 antibody. Our studies did find disrupted membranes attached to mucins, so it is clear that our analysis was not exclusively of intact granules. IgG controls showed little to no background; in fact, most of the misplaced gold-labeled beads were attached to parts of dynal beads that were chipped off during the sectioning preparation.

Once the granule membrane fragments were isolated, proteins associated with these structures then were analyzed by two different techniques. The first of these was Western blotting to identify specific proteins, followed by immunoprecipitation and immunoblotting to probe associations between the proteins. Since we and others have shown previously that MARCKS, HSP70, CSP and hCLCA1 appear to be associated with these membranes [9,27] and play a role in regulated exocytosis [28-31] this analysis was limited to these proteins. As expected, each of these proteins was shown to associate with the isolated granule membrane preparations and with each other (Figure 3).

We then carried out the first (to our knowledge) proteomic LC-MS/MS study of the isolated membranes to determine other proteins that might be associated with the granules. One-dimensional gels coupled with liquid chromatography provide a good separation platform for soluble proteins [13]. Here we excised seven bands and processed them for mass spectrometry analysis. The band sizes were chosen so as not to include MARCKS, hCLCA1 and CSP, based on the expected migration sizes of those proteins, since these proteins were already identified via Western blotting and we were interested in additional, as yet unidentified granule-associated proteins. This is because a "disadvantage" of LC-MS/MS is that signals from proteins of low abundance can be masked by larger, more abundant proteins

What the LC-MS/MS results did reveal, however, was a plethora of cytoskeletal proteins as part of the "granulome", many of which are known to be related to exocytosis and potentially to the mechanisms of mucin secretion in goblet cells. For example, plastins are a class of actin-binding proteins that cross-link actin filaments into tight bundles [32]. Activation of cofilin, a major actin depolymerizing protein, was shown to be necessary for exocytosis in adrenal chromaffin cells [33]. Annexins, a family of calcium-dependent, membrane-associated proteins, are reported to function in endosome sorting, membrane-cytoskeletal linkage and control of fusion events in exocytosis [34]. Annexin A2 phosphorylation has been suggested to be a major regulator of cofilin-dependent actin cytoskeletal dynamics [35]. Gelsolins, actin-binding proteins that regulate actin-mediated movement by controlling assembly and disassembly of actin via severing activity, are upregulated in the bronchial epithelium in asthmatic patients [36,37]. Myosin V is an actin-based molecular motor that functions as "molecular feet", transporting vesicles/organelles from one place to another along actin tracks [38,39]. Furthermore, myosin V facilitates vesicle docking during exocytosis [40]. In the human genome there are three isoforms of Myosin V, myosin-Va, -Vb and -Vc. Recent studies published from this laboratory have shown that Myosin Vc interacts with MARCKS in airway epithelial cells [41].

The regulatory proteins identified as associating with the mucin granule membranes probably did so while acting on other proteins (i.e. PKC, Protein phosphatase 1, Phosphodiesterase 10A). The interaction between actin and myosin is primarily regulated by phosphorylation, and inhibition of the protein phosphatase type 2 (PP2A) inhibited secretion and led to increased phosphorylation of the myosin heavy and light chains at protein kinase C-specific sites in mast cell secretion [42]. Protein phosphatase 1 and 2A dephosphorylate MARCKS in Swiss 3T3 cells and mouse fibroblasts [43] and dephosphorylation of MARCKS via the activity of PP2A is an important component of the airway mucin secretion pathway [7].

Vesicle docking and fusion is regulated by SNAREs (soluble N-ethylmaleimide-sensitive fusion protein attachment protein) receptors of the transport vesicle and target membranes. Syntaxins, as well as VAMPS (vesicle associated membrane proteins), are SNARE proteins essential for exocytosis. It has been shown that Syntaxin 11 facilitates fusion in intracellular membrane trafficking events in lymphocyte-mediated secretion [44].

In conclusion, we have described association of cytoskeletal, regulatory, chaperone and scaffolding proteins with mucin granules in human airway epithelial cells. The process of mucin secretion no doubt occurs as a series of highly cooperative and orchestrated events that culminate with the release of mucin granule contents into the airway lumen. Through the application of proteomic tools we have been able to identify, for the first time in many cases, numerous proteins associated with the granules and probably with the secretory process. Clearly, additional investigations are warranted as to whether or not any of these proteins represent potential therapeutic targets to control excess mucus secretion in different airway inflammatory conditions.

## Competing interests

K.B.A. served on the advisory board for BioMarck, Inc. for less than $1,000, and holds founders shares of stock totaling less than $1,000. He received patents from North Carolina State University for # 6,933,149 B2 Culture system for mouse tracheal epithelial cells and # 7,265,088 B1 Method and composition for altering mucin secretion. He received sponsored grants from the National Institutes of Health and the U.S. Environmental Protection Agency (both for more than $100,001). He also serves as editor-in-chief of the *American Journal or Respiratory Cell and Molecular Biology *and receives a stipend from the American Thoracic Society for this. None of the other authors has a financial relationship with a commercial entity that has an interest in the subject of this manuscript.

## Authors' contributions

KLR performed the experiments and composed the manuscript. KWL assisted with experiments and data. JP, SF and ALC assisted with various phases of the research and contributed to the final manuscript. KBA directed the overall concept, research and resultant manuscript. All authors have read and approved the final manuscript.

## Supplementary Material

Additional file 1 **Table S1**. Mucin granule membrane associated proteins identified by liquid chromatography mass spectrometry (LC-MS/MS). This table contains a listing of proteins discovered with an X-corr value ≥ 2.0. A shortened version of this list appears in the manuscript with X-corr values ≥ 3.0. Click here for file
