# Supplementary material for: Mucin granule-associated proteins in human bronchial epithelial cells: the airway goblet cell "granulome"
Source: Respir Res. 2011 Sep 6;12(1):118. doi: 10.1186/1465-9921-12-118 (PMC3184067; doi:10.1186/1465-9921-12-118)
Supplement: Additional file 1 — Table S1. Mucin granule membrane associated proteins identified by liquid chromatography mass spectrometry (LC-MS/MS). This table contains a listing of proteins discovered with an X-corr value ≥ 2.0. A shortened version of this list appears in the manuscript with X-corr values ≥ 3.0. [file 1465-9921-12-118-S1.PDF]

Table 1. Mucin granule membrane associated proteins identified by liquid chromatography mass spectrometry (LC-MS/MS)

Protein/reference	Xcorr	Protein Accession Number	Charge	Peptide Sequence
MYH9 protein	5.665	29436380	2	K.LQVELDNVTGLLSQSDSK.S
Gelsolin (Amyloidosis, Finnish type)	5.283	4504165	2	-.AQPVQVAEGSEPDGFWEALGGK.-
keratin 1; Keratin-1; cytokeratin 1; hair alpha protein	4.684	17318569	2	-.LNDLEDALQQAK.-
heat shock 70kDa protein 5 (glucose-regulated protein, 78kDa); BiP;	4.516	16507237	2	R.IEIESFYEGEDFSETLTR.A
mitochondrial malate dehydrogenase precursor	4.435	21735621	2	K.VAVLGASGGIGQPLSLLLK.N
bA255A11.8 (novel protein similar to annexin A2 (ANXA2)	4.318	12314197	2	K.SALSGHLETVILGLLK.T
Hypothetical protein DKFZp686P03159	4.318	34364597	2	-.SALSGHLETVILGLLK.-
Cathepsin B (Fragment)	4.3	4505863	2	-.NGPVEGAFSVYSDFLLYK.-
Crystal Structure Of Human Recombinant Procathepsin B At 3.2 Angstrom Resolution	4.3	2982114	2	K.NGPVEGAFSVYSDFLLYK.S
anterior gradient 2 homolog (Xenepus laevis) , agr2	4.116	30583839	2	-.LAEQFVLLNLVYETTDK.-
mutant beta-actin (beta'-actin)	4.084	28336	2	K.SYELPDGQVITIGNER.F
Annexin A2	3.904	16306978	2	R.RAEDGSVIDYELIDQDAR.D
ARC16-2	3.761	33150554	2	-.ALAVGGLGSIIR.-
Hypothetical protein DKFZp686E1899	3.632	57997483	2	-.QLETVLDDLDPENALLPAGFR.-
tropomodulin 3	3.632	6934244	2	K.QLETVLDDLDPENALLPAGFR.Q
PRO1708	3.611	7959791	3	-.RHPDYSVVLLLR.-
similar to beta-actin	3.502	37546764	2	-.SYKLPDGQVITIGNER.-
calmodulin 1 (phosphorylase kinase, delta)	3.458	30583815	2	R.VFDKDGNGYISAAELR.H
A Chain A, Human Serum Transferrin, Recombinant N-Terminal Lobe, Apo Form	3.41	4389230	2	-.DQYELLCLDNTR.-
Nuclease sensitive element binding protein-1	3.374	34098946	2	-.SVGDGETVEFDVVEGEK.-
Actin-like protein (Fragment)	3.294	62421184	2	-.VAPEEHPVLLTQAPLNPK.-
uracil DNA glycosylase	3.284	35053	2	-.LVINGNPITIFQER.-
Myosin regulatory light chain MRCL3 variant (Fragment)	3.259	62896697	2	-.ATSNVFAMFDQSQIQEFK.-
Keratin, type II cytoskeletal 3 (Cytokeratin 3) (K3) (CK3)	3.206	125098	2	-.FLEQQNKVLETK.-
unnamed protein product	3.19	28940	2	-.FTQAGSEVSALLGR.-
Unknown (protein for MGC:71545)	3.1	37747855	2	-.NLNEKDYELLCLDGTR.-
Hypothetical protein DKFZp451J0218	3.063	30268331	2	-.VSHLLGINVTDFTR.-
keratin 19; keratin, type I cytoskeletal 19; keratin, type I, 40-kd;	3.024	24234699	2	R.GQVGGQVSVEVDSAPGTDLAK.I
pyruvate dehydrogenase (lipoamide) beta; Pyruvate dehydrogenase, E1 beta	2.97	4505687	2	K.VFLLGEEVAQYDGAYK.V

Table 1 (continued)

Protein/reference	Xcorr	Protein Accession Number	Charge	Peptide Sequence
similar to hypothetical protein FLJ20420	2.903	37547262	2	-.RVAEELALEQAK.-
ASC-1 complex subunit P200	2.826	12061185	3	K.VKQCVHQIPSVMMEASIQPITR.T
keratin 4; Keratin-4; cytokeratin 4; keratin, type II cytoskeletal 4	2.815	17318574	2	-.NLDLDSIIAEVR.-
V_segment translation product	2.767	1552502	3	.MSTRLLCWMALCLLGAELSEAEVAQSPRYK.
pyruvate kinase M2	2.75	6018096	2	R.LAPITSDPTEATAVGAVEASFK.C
FLJ21945 protein	2.744	23272708	3	-.GICFLTDQLLLILVGKQK.-
Mitochondrial ornithine transporter 2	2.71	38372886	3	-.NEGIVALYSGLKATMIR.-
CLIP-associating protein CLASP1	2.67	13508645	3	-.SGNMIQSANDKNFDDEDSVDGNR.-
cytokeratin type II	2.631	4758618	2	-.LVDLEEALQKAK.-
E1A binding protein p400; p400 SWI2/SNF2-related protein; CAGH32 prote	2.63	15805014	3	-.RVLILSQMILMLDILEMFLNFHYLTYVR.-
TATA element modulatory factor 1	2.599	6005904	3	-.YQVELENLKDEYVRTLEETR.-
hypothetical protein FLJ14721	2.57	14249534	3	R.YPSPAELDAYAEKVANSPLSIKIFPTNIR.V
ras homolog gene family, member C; Aplysia RAS-related homolog 9	2.568	28395033	2	K.QVELALWDTAGQEDYDR.L
Integrin alpha-D precursor (Leukointegrin alpha D) (CD11d) (ADB2)	2.524	12643717	3	-.KPPQHSDFLTQISR.-
C11orf15 protein	2.523	31455206	3	-.RLFGHAQLIQSDDDIGDHQPFANAHDVLAR.-
protein kinase C-like 2	2.511	5453974	3	-.LEELHHKLQELNAHIVVSDPEDITDCPR.-
similar to cytoplasmic beta-actin	2.45	29736622	2	-.VAPDEHPILLTEAPLNPK.-
Hypothetical protein KIAA0143	2.449	2495710	3	SNGRHGAVGAPCAAPLSLGAAASAVEIAMPT
inhibitory receptor IREM1	2.447	31790204	3	-.LSSAQVDQVEVEYVTMASLPK.-
fragile X mental retardation syndrome related protein 2; fragile X-ment	2.436	4758410	3	R.TDEDRTVMDGGLESDGPNMTENGLEDESR.
unnamed protein product	2.436	34526743	2	R.SACATRQNSTSTKNTK.I
zinc finger protein 132 (clone pHZ-12)	2.436	4507979	3	-.PYSNLGQLPEVCTTQKLFECSNCGKAFLK.-
similar to nuclear pore complex interacting protein	2.434	37541436	3	-.KSAVQQLTPLLLR.-
CD1A protein	2.425	21594951	2	-.FILGLLDAGKAHLQR.-
Membrane alanine aminopeptidase precursor	2.415	37590640	3	-.YLSYTLNPDLIR.-
C59436 KIAA1391 protein [imported]	2.41	25535895	2	-.YINLEKEKDYPK.-
solute carrier family 24 (sodium/potassium/calcium exchanger), member 1	2.405	4759128	3	R.QKQAIYLFLLPIVFPLWLTVPDVR.R
low density lipoprotein-related protein 1B; low density lipoprotein rec	2.404	9055270	3	R.AWDTLYWTSSTTSSITRHTVDQTR.P
parkin isoform	2.403	20385800	3	-.EPQSLTRVDLSSSVLPGDSVGLAVILHTDSR.-
KIAA0713 protein	2.402	3882147	2	-.AQQKITEKDDQVK.-

Table 1 (continued)

Protein/reference	Xcorr	Protein Accession Number	Charge	Peptide Sequence
Unknown (protein for MGC:71515)	2.389	34785925	3	-.LLQQNVDICAEATCGMIAER.-
Thyroid hormone receptor interactor 6	2.385	12803689	3	-.EPGPGAKEEAAGISGPAGR.-
T00095 hypothetical protein KIAA0470	2.372	7512995	3	-.EDNKTDEGPDTPSYNR.-
SH3-domain binding protein 5 (BTK-associated); SH3 binding protein	2.368	4759058	3	-.TRSELVHKETAAR.-
hypothetical protein	2.366	12052938	3	K.GQAAPPAPPLPSSLDPPPPPAAVEVFQR.P
similar to Hypothetical protein KIAA0056	2.359	20481728	2	-.MVALRGLGSGLQPWCPLDLR.-
phosphodiesterase 10A	2.354	5729972	2	-.VIRGEETATWISSPSVAQK.-
unnamed protein product	2.354	30846	3	-.QAEGLSEDGAAMAVEPTQIQLSKR.-
unnamed protein product	2.336	34533353	3	.CLPVERRCDGLQDCGDGSDEAGCPDLACGR.
Similar to KIAA0052 protein	2.32	21619317	2	-.LYIPKDLRPVDNR.-
unnamed protein product	2.32	7022460	3	K.PLGLKDCIIVGGMDMVAQALELSR.K
unnamed protein product	2.318	10440008	3	-.MRPKVMWHLLR.-
similar to RIKEN cDNA 5031434M05	2.317	37540268	2	-.FCGLFMVLLSDHPSPDLGQK.-
RB1CC1 protein	2.314	17028471	3	-.VDSAMETSMMSVQENIHMLSEEKQR.-
unnamed protein product	2.305	14042913	2	R.FPLNGYCRLNSVQVLER.L
unnamed protein product	2.303	34531680	3	-.MPKGGCPKAPQQEELPLSSDMVEK.-
similar to DKFZP434P1750 protein	2.297	37541041	2	-.LALGTAEQR.-
similar to hypothetical protein FLJ31547	2.291	29746676	3	-.AIPSIIGVNNPECGFPLPMKEAPEILSGSNK.-
ubiquitin associated protein 2 isoform 2; AD-012 protein	2.29	22325366	3	-.SSYGLKGAWKNSVEEWTTEDWTEDLSETK.-
plastin 3; T isoform	2.283	7549809	2	-.PPYPKLGANMKK.-
breast carcinoma amplified sequence 1; Breast carcinoma amplified sequence	2.276	4502373	3	-.VDEVPGLSGQSDDVPAGKDIVDGKEK.-
stromal cell derived factor receptor 1 isoform b	2.272	6912646	3	K.NEGQDATMYCKSVGYPHPDWIWRK.K
Sec3-like isoform 1; homolog of yeast exocyst protein Sec3p; exocyst c	2.26	30410720	2	R.ELQVLDGANIQSIMASEK.Q
NACHT, LRR and PYD containing protein 10	2.258	28827807	3	-.KSQSQNLFSVKSSLSHGPK.-
transducer of regulated CREB protein 3	2.241	37693045	3	-.QQPPWKDEKHPGFR.-
chromosome 14 open reading frame 50	2.239	27312029	3	K.RLGGQTPYLMDQLGLRLGMWYWK.D
ubiquitin specific protease 32	2.233	28188773	3	-.NKDMSWPEEMSFIANSSKIDR.-
hypothetical protein	2.232	12053087	3	R.DVTFAQEFINLDGISLLTQMVESGTER.Y
unnamed protein product	2.232	34530583	3	.ALMNEKAQAALVEFVEDVNHAAIPREIPGK.I
unnamed protein product	2.228	10437181	3	-.SPDLYERQVCLLLLQLCSGLEHLK.-

Table 1 (continued)

Protein/reference	Xcorr	Protein Accession Number	Charge	Peptide Sequence
neuroblastoma-amplified protein	2.226	22164066	2	-.MLSVKFYSRQGQEQDGIFK.-
similar to Nck-associated protein 5 (NAP-5)	2.211	37547356	2	-.SGRLLFFARNYSVTTATR.-
RTKN protein	2.21	33871180	2	-.GRVCISDLRIPLMWK.-
dystrophin Dp116 isoform	2.208	5032295	3	-.TYHVKDLQGEIEAHTDVYHNLDENSQK.-
prostaglandin E receptor 4, subtype EP4; PGE receptor, EP4 subtype	2.204	4506259	3	-.NPDLQAIRIASVNPILDPWIYILLRK.-
A Chain A, Determination Cc-Chemokine Mcp-3, Nmr, 7 Structures	2.202	2624707	2	-.EAVIFKTKLDK.-
similar to soluble adenylyl cyclase	2.202	37552182	3	K.SSLCWFSREGLLATAQLMQALAYTK.L
similar to surface glycoprotein, Ig superfamily member	2.202	37541781	2	-.LVFQQLKRSQTK.-
MY9B_HUMAN Myosin IXb (Unconventional myosin-9b)	2.199	14548118	3	K.SPQVPRDIQEEELEVLLEEEAAGGDEDREK.E
KIAA0614 protein	2.192	34328016	3	K.ACNAHGGVFKDEIYIPLQEEDTK.K
R33083_1	2.191	3702295	3	-.PCGGKEFGLFEELSEGSFGWVTGIRR.-
LYST-interacting protein LIP8	2.19	29789293	3	-.PLQDLSPSSSAQALEELFPRYTSLR.-
SI:zC214P16.4 (novel protein similar to human protein phosphatase 1)	2.186	27884151	3	-.IMRPTDVPDTGLLCDLLWSDPDK.-
hypothetical protein	2.184	31873973	3	-.MVHSLPTAVPESPRIHPTRTPK.-
MTMR15 protein	2.181	28839601	3	-.EECIPEHMVRESKIMEAESQK.-
DKFZp434C0631 protein	2.179	28279320	2	K.NDHVLFYLENVFGR.A
Src substrate cortactin (Amplaxin) (Oncogene EMS1)	2.178	2498954	2	-.ELETGPKASHGYGGK.-
unnamed protein product	2.174	16549252	2	-.TCQVDQFSCGNGR.-
amylo-1,6-glucosidase, 4-alpha-glucanotransferase isoform 1; glycogen	2.172	4557275	3	-.WNPEALPSNTGEVNFQSGIIAARCAISK.-
hypothetical protein	2.172	30268329	2	-.EVMCQLGLHQK.-
hypothetical protein MGC46732	2.168	24308460	3	-.LKEESLSLFTILHDRILEIEK.-
similar to hypothetical protein	2.167	37541409	3	-.FYKPFNLMFYQMTGQDFQWEQEEGDK.-
dJ469A13.3 (continues in Em:AL031662 as dJ460J8.1)	2.162	13277305	2	-.PLLNDKNGTRNFQDFDCQ.-
syntaxin 11	2.157	3248918	3	-.WDVFSENLLADVKGR.-
cofilin 1 (non-muscle)	2.154	5031635	3	-.EILVGDVGQTVDDPYATFVKMLPDK.-
A-kinase anchor protein 3 (Protein kinase A anchoring protein 3)	2.153	14194457	2	-.SCDASLAELGDDKSGDASR.-
calreticulin precursor; Sicca syndrome antigen A	2.15	4757900	3	K.SGTIFDNFLITNDEAYAEEFGNETWGVTK.A
NDRG2 protein (Syld709613 protein)	2.148	20141615	3	-.TASLTSAASVDGNRSRSR.-
KIAA0550 protein	2.142	20521081	3	K.ELDESSVFVLGAVLYKNLDLILPTLR.N
transmembrane channel-like protein 7	2.141	33355703	3	R.IPSSKACGPFTNFNTTWEVIPK.T

Table 1 (continued)

Protein/reference	Xcorr	Protein Accession Number	Charge	Peptide Sequence
caspase 14 precursor; apoptosis-related cysteine protease	2.139	6912286	3	-.FQQAIDSREDPVSCAFVVLMAHGR.-
microtubule-associated protein 1B isoform 2	2.135	14165456	3	-.HPDVSMVDPEALAIEQNLGKALK.-
breast cancer antiestrogen resistance 3	2.133	4502371	3	-.NMPVNHQFPLASSMDLLSSRSPLAEHR.-
unnamed protein product	2.133	14042842	3	R.RASVKACIANVEPNQTVEINEQEALEEK.L
IKK-related kinase epsilon; IKK-related kinase epsilon; inducible Ikapp	2.131	7661946	2	R.DQVHEDRSIQQIQCCLDK.M
myosin VC; myosin 5C	2.127	9055284	2	-.SGSNAHVEDKVLASNPITEAVGNAK.-
neutral alpha glucosidase C type 2	2.126	25272050	3	-.STYQALLDSVTTDEDSTRFQIINEASK.-
hypothetical protein FLJ14871	2.123	14249582	3	R.RSQSASDAPLSQHTLETLLEEIKALR.E
Unknown (protein for IMAGE:3838106)	2.119	13325406	3	[illegible].FNQCYGVLGVLDHLHGTDTMFKQTKAYER.[illegible]
hypothetical protein MGC39518	2.118	28376658	3	-.VIQDSNNELLEPVCHQLFELYRSSEVR.-
unnamed protein product	2.116	10434173	3	-.QHILPAEKEVTEFYVQNENSVDKWGK.-
unnamed protein product	2.115	28071062	3	-.MGIGTGHTSMNKGGKDVTLLELSVEK.R
FKSG16	2.112	16416764	2	-.VPFLFTIVPFSVLR.-
MGC4707 protein	2.109	15559265	3	-.NGDLLTMKEYHCLLQLLCPDFPLELTQK.-
ash1 (absent, small, or homeotic)-like	2.107	8922081	3	-.TLGKPDSLLVPAVASDSCNNSISLLSEK.-
ADCY7 protein	2.106	25058301	3	-.HQAILGMAFLVLAVFAALSVLMYVECLLR.-
KIAA1729 protein	2.104	12698003	3	K.SLTVDGNVGKLVGIDSFQPSVQK.Q
FLJ00056 protein	2.102	10440440	3	-.YGRLLEELLREAGPELSSECR.-
cytokine receptor-like factor 1	2.092	4758062	3	R.GPAAQSARRPPPLLPLLLLLCVLGAPR.A
ribosomal protein P0	2.092	4432757	2	R.VLALSVETDYTFPLAEK.V
RPLP0 protein	2.092	75771977|	2	-.VLALSVETDYTFPLAEK.-
ankyrin repeat and FYVE domain containing 1 isoform 2; ankyrin repeat	2.091	31317252	3	[illegible].HSADVMSEMAQIAEALLQAGANPNMQDSK.[illegible]
AGLW2560	2.09	37182360	2	-.RSSWSATGSAAPFPPSDQPGTR.-
alpha-catenin-like protein	2.083	7019571	2	-.SNTLNIALDNMCKK.-
KPI-2 protein	2.083	27356940	3	-.KVFPLRWTAPELVTSFQDR.-
nebulin	2.079	4758794	3	-.INIPADMVSVLAAK.-
seven transmembrane helix receptor	2.075	21928245	3	-.SVTPPPFISPTSQLVITFSLTSLQESVTFR.-
KIAA0376	2.074	2280485	3	-.SFDSASQVPNPAAAAIPR.-
citron; rho-interacting, serine/threonine kinase 21	2.071	32698688	2	R.SDLYESELRESRLAAEEFK.R
hypermethylated in cancer 1	2.071	5729871	3	-.IHSGEKPYECQVCGGKFAQQR.-

Table 1 (continued)

Protein/reference	Xcorr	Protein Accession Number	Charge	Peptide Sequence
KIAA1200 protein	2.065	6330407	3	K.VGTAYEQLIGKLMDGEGDPDSPLWR.H
SH3-domain kinase binding protein 1; c-Cbl-interacting protein	2.058	13994242	3	-.PPSQSLTSSSLSSPDIFDSPSPEEDK.-
protein phosphatase 4, regulatory subunit 2	2.057	28372531	3	K.KEVCPVLDQFLCHVAKTGETMIQWSQFK.G
ubiquitin specific protease, proto-oncogene; Unph	2.057	4507853	3	-.PDAETQKSELGPLMR.-
MAGUK p55 subfamily member 6 (VELI-associated MAGUK 1) (VAM-1)	2.054	27764857	3	R.TSEFMPYVVFIAAPELETLRAMHK.A
KIAA1082 protein	2.052	14133233	2	K.GIPEHLMGKLGPNGER.S
A44256 zinc-finger protein ZNF76 - human	2.051	2136421	3	-.THTGERPFQCPFEGCGRSFTTSNIR.-
KIAA1950 protein	2.049	18916759	3	R.ALKDALVSTDAALQQLYVSAFSPAER.L
phosphatidylinositol 3-kinase	2.047	987948	2	K.AHRQGHMVKVDWLDR.L
unnamed protein product	2.046	21754782	2	K.HIDLLKIKVTSGVGDK.Q
similar to 16.7Kd protein	2.045	37549528	3	K.QFLECAENQGDIKLCEGFNEVLK.Q
T08775 hypothetical protein DKFZp586C1620.1	2.044	7512857	3	-.EDFFHCLKCNLCLAMNLQGRQVY.-
hypothetical protein MGC45962	2.042	22749313	3	-.MLVIAGGILAALLLLIVVVLCLYFKIHNALK.-
signal peptidase complex 18 kDa subunit	2.042	3641344	2	-.ARGFVPYIGIVTILMNDYPKFR.-
PIP3AP protein	2.041	34783714	3	R.EFYDSWHSKSTDYHGLLLPHIEGPEIK.V
FLJ00141 protein	2.035	18676488	3	-.LSTAITLLPVEEGR.-
XPA binding protein 2; XPA-binding protein 2	2.033	9910260	3	-.ALKLLPCSTKLWYR.-
KIAA0286	2.031	20521027	3	-.PVPPRLLTEEEYRIQGEVETR.-
capacitative calcium entry channel protein; identical to AJ006203	2.029	3264578	3	-.HWVVKLLTCMTIGFLFPMLSIAYLISPR.-
unnamed protein product	2.028	34528602	3	-.AQTHPAPPQTWAPFFTMHSSEQPCR.-
microtubule-based motor	2.027	4106715	3	R.SHAVFNIIFTQKRHDAETNITTEK.V
IGF-II mRNA-binding protein 1	2.026	21361352	3	-.LLVPTQYVGAIIGKEGATIRNITK.-
DEAD-box protein abstrakt [synthetic construct]	2.023	30584005	3	-.VPPVLQVLHCGDESMLDIGGER.-
KIAA1564 protein	2.018	34328020	2	-.WCSLPYEDSTWELKEDVDEGK.-
KIAA1196 protein	2.016	34013528	3	K.AENQALRDIPLSLMNDWKDEFK.A
ADAM 2 precursor (A disintegrin and metalloproteinase domain 2)	2.012	28202251	2	-.VCRNQRCVSSSYLGYDCTTDK.-
cyclin D-dependent kinases 4 and 6-binding protein/p16 product	2.012	861472	2	-.GSNHARIDAAEGPSAIPD.-
unnamed protein product	2.01	10435867	3	-.QWLSATKPPLSDR.-
hypothetical protein XP_352404	2.007	37560019	3	R.ALYTTLLMIPTRHANVDAVHDIANEDTV.-
very-long-chain acyl-CoA dehydrogenase	2.007	3273228	2	-.ALEQFATVVEAK.-
